# Inverted *Alu* repeats: friends or foes in the human transcriptome

**DOI:** 10.1038/s12276-024-01177-3

**Published:** 2024-06-14

**Authors:** Keonyong Lee, Jayoung Ku, Doyeong Ku, Yoosik Kim

**Affiliations:** 1 Department of Chemical and Biomolecular Engineering, Korea Advanced Institute of Science and Technology (KAIST), Daejeon, 34141 Republic of Korea; 2 Graduate School of Engineering Biology, KAIST, Daejeon, 34141 Republic of Korea; 3 KAIST Institute for BioCentury (KIB), Daejeon, 34141 Republic of Korea; 4 KAIST Institute for Health Science and Technology (KIHST), Daejeon, 34141 Republic of Korea; 5 BioProcess Engineering Research Center and BioInformatics Research Center, KAIST, Daejeon, 34141 Republic of Korea

**Keywords:** Long non-coding RNAs, RNA metabolism

## Abstract

*Alu* elements are highly abundant primate-specific short interspersed nuclear elements that account for ~10% of the human genome. Due to their preferential location in gene-rich regions, especially in introns and 3′ UTRs, *Alu* elements can exert regulatory effects on the expression of both host and neighboring genes. When two *Alu* elements with inverse orientations are positioned in close proximity, their transcription results in the generation of distinct double-stranded RNAs (dsRNAs), known as inverted *Alu* repeats (IR*Alus*). IR*Alus* are key immunogenic self-dsRNAs and post-transcriptional *cis*-regulatory elements that play a role in circular RNA biogenesis, as well as RNA transport and stability. Recently, IR*Alus* dsRNAs have emerged as regulators of transcription and activators of Z-DNA-binding proteins. The formation and activity of IR*Alus* can be modulated through RNA editing and interactions with RNA-binding proteins, and misregulation of IR*Alus* has been implicated in several immune-associated disorders. In this review, we summarize the emerging functions of IR*Alus* dsRNAs, the regulatory mechanisms governing IR*Alus* activity, and their relevance in the pathogenesis of human diseases.

## Unlocking Alu elements: primate-specific genomic regulators unveiled

Understanding the role of *Alu* elements—short DNA sequences scattered throughout our genome—is crucial for grasping human genetics. These elements can affect how our genes function, and sometimes, their misregulation causes diseases. However, there’s still much we don’t know about their impact on gene regulation and cell signaling, including innate immune responses. Lee et al. summarized how *Alu* elements interact with our immune system by generating specific structures similar to ones from viruses. The authors suggest that further understanding of biological processes regulated by *Alu* elements could lead to advancements in the development of potential treatment for diseases linked to immune system dysfunction. Future research could explore how to manipulate these elements to benefit human health.

This summary was initially drafted using artificial intelligence, then revised and fact-checked by the author.

## Introduction

*Alu* elements are ~300 base-pair (bp) long primate-specific short interspersed nuclear elements (SINEs) that constitute approximately 10% of the human genome^1^. They are originated from the fusion of two distinct short arms of 7SL RNA-derived sequences linked by an A-rich region^2^. They contain an internal RNA polymerase III (Pol III) promoter, which can initiate independent transcription^3^ and contribute to *Alu* amplification in the genome with the assistance of autonomous long interspersed nuclear elements, such as ORFp2^4^. Throughout evolution, *Alu* retrotransposition has given rise to new copies with distinct mutations, leading to the formation of diverse subfamilies characterized by sequence variations and relative ages^2^.

Due to their high abundance and insertion preference in gene-rich regions, *Alu* elements can regulate gene expression at the transcriptional, post-transcriptional, and even translational level^5^. *Alu* insertions provide locations for DNA methylation and other epigenetic modifications, as *Alu* sequences are responsible for approximately 25% of CpG dinucleotides in the human genome^6^. Moreover, *Alu* elements can act as enhancers and activate the transcription of nearby genes by facilitating 3-dimensional long-range chromosomal interactions^7^. When transcribed, *Alu* RNAs impose additional gene regulatory effects independent of *Alu* amplification. During the heat shock response, *Alu* RNAs are transcribed by Pol III and can act as *trans*-acting transcriptional repressors by directly binding to Pol II and blocking transcription at the initiation step^8^. Interestingly, *Alu* elements embedded in introns and 3′ UTRs are transcribed as a part of the host mRNAs and serve as key post-transcriptional regulatory elements, with alternative splicing being a well-established biological process that is regulated by *Alus*. In this context, *Alu* sequences provide multiple potential splicing donor and acceptor sites for differential usage of splice sites of the host mRNAs^9^. Finally, *Alu* insertions in UTRs are known to modulate the translation of host genes by affecting the rate of initiation^5,10–12^.

**Fig. 1:**
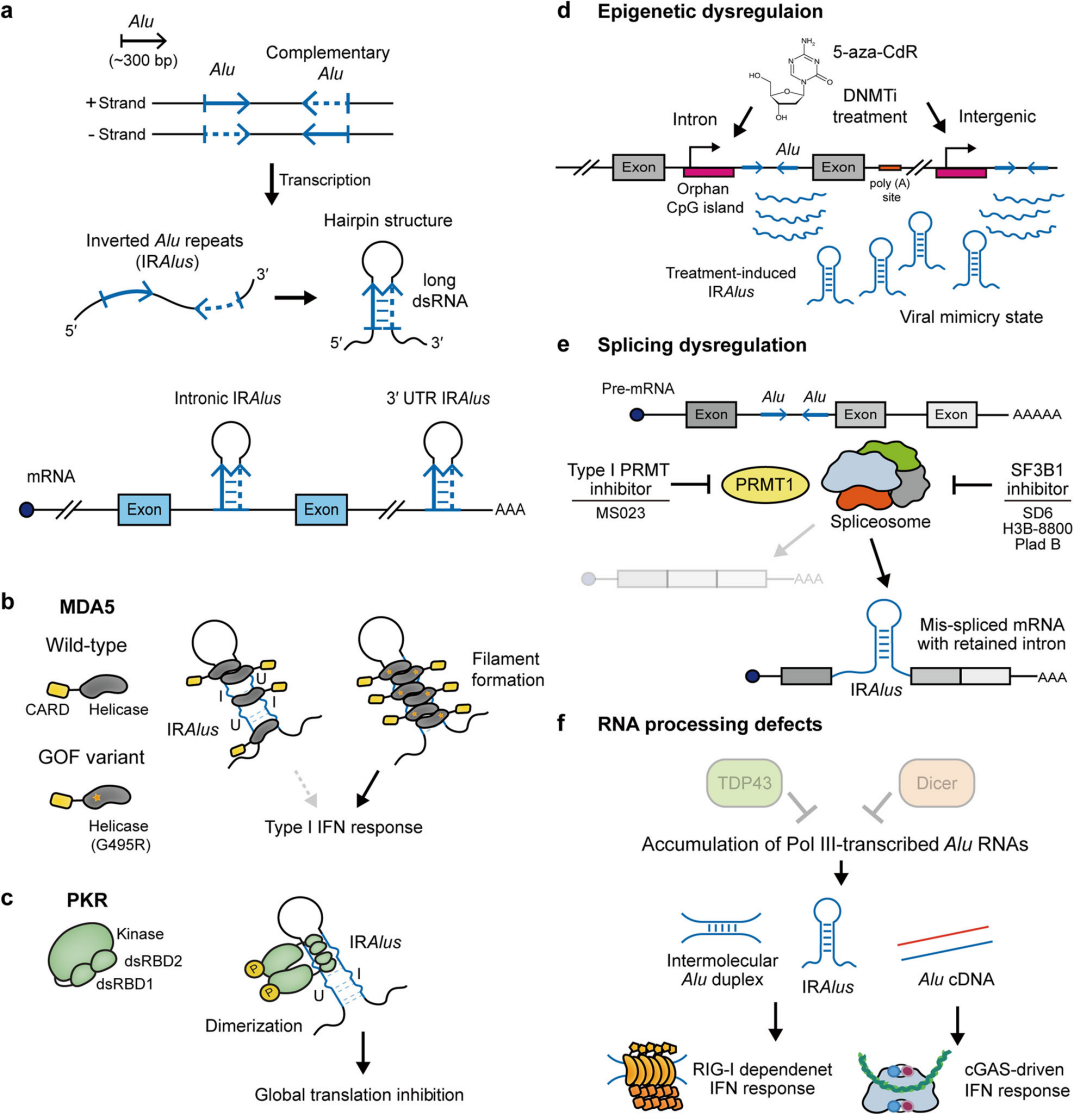
IR*Alus*-mediated immune response. **a** Two *Alu* elements with opposite directions can be present in a single gene. When transcribed, these *Alu* elements can form an intramolecular dsRNA structure, referred to as IR*Alus*. These IR*Alus* are predominantly found within introns and 3′ UTRs. **b**, **c** Recognition of IR*Alus* by dsRNA sensors. **b** The gain-of-function mutant MDA5 (G495R) undergoes aberrant oligomerization via robust sensing of IR*Alus*, which subsequently triggers an IFN response, whereas wild-type MDA5 does not normally recognize IR*Alus*. **c** PKR is activated by IR*Alus* and suppresses global translation. **d** Epigenetic dysregulation via a DNMT inhibitor (DNMTi) triggers cryptic transcription of intronic and intergenic IR*Alus*, inducing a viral mimicry state. **e** Disturbance of splicing by a PRMT inhibitor or spliceosome inhibitor induces the accumulation of mis-spliced mRNAs that retain intronic IR*Alus* and activate the I-IFN response. **f** RNA processing defects caused by TDP-43 or Dicer deficiency result in the accumulation of Pol III-transcribed *Alu* RNAs. These unprocessed *Alu* RNAs initiate the formation of both *Alu* duplex RNAs and *Alu* cDNAs, leading to the activation of RIG-I-dependent and cGAS-driven IFN responses, respectively.

One special feature of *Alu* elements is provided by their sequence similarities and abundant copy numbers. A single gene typically contains multiple *Alu* elements with positive and negative orientations^13,14^. When transcribed, two neighboring *Alus* elements with opposite orientations can fold back and form an intramolecular hairpin structure with a long double-stranded stem (Fig. 1a). Known as inverted *Alu* repeats (IR*Alus)*, IR*Alus* constitute an important class of endogenous double-stranded RNAs (dsRNAs) in human cells^14^. Originally, dsRNAs were considered hallmarks of viral infection because they are produced during viral transcription or replication^15^. In vertebrates, viral dsRNAs are recognized by pattern recognition receptors (PRRs), including protein kinase R (PKR) and melanoma differentiation-associated protein 5 (MDA5), and activate antiviral signaling pathways^16^. Recent studies have shown that endogenous dsRNAs, including IR*Alus*, can also activate PRRs and induce a viral mimicry state in uninfected cells^17–19^. Consequently, IR*Alus* are closely associated with immune-related diseases, and modulating IR*Alus* expression has been suggested as a potential therapeutic strategy for cancer^19–21^. In addition to the well-established immunogenic role, IR*Alus* participate in post-transcriptional gene regulation through distinct mechanisms of action. Intronic IR*Alus*, which account for almost half of all IR*Alus*, are strongly associated with the generation of thousands of circular RNAs (circRNAs) by facilitating the backsplicing of exons^22^. When IR*Alus* are located in the 3′ UTR, they suppress gene expression by inhibiting cytosolic export of host mRNAs, sequestering them in nuclear paraspeckles^13,23^. Therefore, properly regulating IR*Alus* activity is important, and to do so, cells employ various dsRNA-binding proteins (dsRBPs) that recognize the double-stranded secondary structure of IR*Alus*^20,24–27^. In this review, we aim to summarize recent findings on the multifaceted role of IR*Alus* and establish IR*Alus* as an essential gene regulatory element in human pathophysiological conditions.

### Innate immune activation by IR*Alus*

Human dsRNAome captured using an anti-dsRNA J2 antibody by Kim et al. revealed that IR*Alus* are the most abundant class of cellular dsRNAs^17^. Furthermore, genome-wide sequencing analysis indicated that adenosine-to-inosine (A-to-I) editing, which occurs within dsRNAs longer than 50 bp, is observed primarily on IR*Alus*^28^. More importantly, an increasing number of reports suggests a close association of self-derived dsRNAs in the abnormal immune activation behind the pathogenesis of immune-related diseases, highlighting the importance of IR*Alus* regulation in cell pathophysiology^14^. In this section, we describe the immunogenic function of IR*Alus* as substrates for dsRNA sensors and share recently uncovered cellular contexts that can modulate IR*Alus*-mediated antiviral responses, including epigenetic and splicing dysregulation.

#### IR*Alus*: immunogenic self-dsRNAs

Our understanding of IR*Alus* that act as immunogenic self-dsRNAs has been significantly expanded by profiling substrates of dsRNA-recognizing immune sensors, including MDA5 and PKR. When bound to long dsRNAs, MDA5 undergoes filament assembly along the RNA and recruits adapter mitochondrial antiviral signaling (MAVS) proteins to induce a downstream type I interferon (I-IFN) response^29^. Ahmad et al. developed an MDA5 protection assay to identify endogenous RNA agonists of MDA5 in the cytosol^19^. The main idea was that a filament of MDA5 along the dsRNA would protect the RNA from RNases. Through such a strategy, the authors revealed that the IR*Alus* located in the 3′ UTR of mRNAs serve as primary ligands of MDA5. Intriguingly, wild-type MDA5 has limited ability to recognize IR*Alus* and inefficiently forms the filaments, compared to gain-of-function (GOF) mutant MDA5, which has enhanced RNA binding affinity and is often found in patients with inflammatory diseases, including Aicardi–Goutières syndrome (AGS)^30^. Indeed, robust recognition of IR*Alus* by the mutant MDA5 promotes aberrant oligomerization of MDA5 and subsequent activation of the I-IFN response (Fig. 1b).

PKR is another innate immune protein that becomes activated upon binding to IR*Alus*^17,18^. To identify PKR-interacting endogenous dsRNAs, Kim et al. performed formaldehyde-mediated crosslinking and immunoprecipitation sequencing (fCLIP-seq) analysis^17^. The authors utilized formaldehyde to crosslink the dsRNA-RBP complex to overcome the low crosslinking efficiency of UV light on dsRNAs^31^. The authors found that more than 20% of the dsRNAs that interact with PKR are derived from *Alu* repeats, nearly all of which are IR*Alus* (Fig. 1c). In a recent study, intronic and intergenic inverted transposable elements (TEs), including IR*Alus*, were found to accumulate upon the inhibition of nuclear RNA decay by phosphorothioate DNAs. The increased expression of these inverted TEs resulted in the activation of dsRNA-sensing pathways, thereby providing additional evidence that IR*Alus* can activate PKR^32^. Overall, high-throughput sequencing methods tailored to study dsRNAs showed that IR*Alus* are the prominent source of immunogenic self-dsRNAs that activate multiple dsRNA sensors.

#### Dysregulation of *Alu* homeostasis and IR*Alus*-mediated aberrant innate immune responses

Due to their immunogenic activity, IR*Alus* generation is suppressed via multiple mechanisms. In most differentiated cells, TEs are subjected to transcriptional silencing through epigenetic mechanisms involving DNA methylation and histone modifications^33,34^. As a result, treating cells with 5-aza-2′-deoxycyctidine (5-Aza-CdR), a DNA methyltransferase inhibitor (DNMTi), derepressed the transcription of TEs and activated antiviral signaling pathways due to increased expression of self-dsRNAs^35,36^. In addition, in a recent study, the authors performed the MDA5 protection assay to identify drug-induced TEs; the results revealed enrichment of *Alus*^20^. Moreover, these drug treatment-induced *Alus* were mostly IR*Alus* originating from intronic and intergenic regions downstream of CpG islands. Intriguingly, some *Alus* adopted an intramolecular hairpin pairing with adjacent *Alus* within a single transcript rather than the previously suggested model of hybridization between sense and antisense transcripts. This observation indicated that inhibiting DNA methylation triggers cryptic transcription of IR*Alus*, which results in the formation of dsRNAs that stimulate MDA5 activation (Fig. 1d).

A substantial portion of *Alu* elements can be expressed from introns during the transcription of their host genes. Consequently, the dysregulation of mRNA splicing is closely associated with antiviral responses. Depletion of heterogeneous nuclear ribonucleoprotein C (hnRNPC) leads to increased dsRNA levels derived from *Alu* elements within pre-mRNA introns, thereby activating the dsRNA sensor retinoic acid-inducible gene I (RIG-I) and eventually inhibiting cell proliferation^37^. In addition, inhibiting type I protein arginine methyltransferase (PRMT) with a small molecule MS023 can upregulate the expression of intronic IR*Alus*^38^. Type I PRMTs are responsible for the asymmetric demethylation of proteins and can control RNA splicing and DNA repair^39^. In this context, inhibiting type I PRMTs alters mRNA splicing, which results in increased intron retention in triple-negative breast cancer cells. IR*Alus* embedded within retained introns of mis-spliced mRNAs activate RIG-I and Toll-like receptor 3 (TLR3) to facilitate cell death^38^. Together, these findings reveal intronic IR*Alus*-mediated innate immune activation within the context of splicing dysregulation (Fig. 1e).

Like those used for PRMT1 inhibition, small chemical inhibitors of the spliceosome have been employed in cancer therapy to induce viral mimicry through increased expression of intronic dsRNAs. Bowling et al. demonstrated that small spliceosome modulators, such as sudemycin D6 (SD6) and H3B-8800, led to widespread cytosolic accumulation of mis-spliced mRNAs, many of which adopt double-stranded secondary structures^21^ (Fig. 1e). These RNAs are recognized by dsRNA sensors, which trigger antiviral signaling and extrinsic apoptosis. Similarly, pharmacological modulation of pre-mRNA splicing factor 3b subunit 1 (SF3B1) using pladienolide B (Plad B) leads to aberrant production of intron-retained mRNAs, thereby activating the RIG-I-dependent I-IFN response and facilitating tumor cell death^40^. These studies highlight the fact that activating innate immune responses by IR*Alus* through DNMTis and spliceosome inhibitors can provide clinical benefit in cancer by inducing a viral mimicry state, suggesting that IR*Alus* are potential therapeutic targets for cancer immunotherapy.

Antiviral responses induced by IR*Alus* can be modulated through direct interaction with RBPs, such as TAR DNA-binding protein 43 (TDP-43) and Dicer. TDP-43 plays an essential role in mRNA metabolism, including transcription, splicing, stability, and transport^41,42^. Accumulating evidence has suggested that the loss of TDP-43 results in the accumulation of self-dsRNAs, including Pol III-transcribed *Alu* RNAs^43,44^. An increase in the cytosolic level of *Alu* RNAs is recognized by RIG-I and induces IFN-mediated cell death (Fig. 1f). Notably, the *Alu* consensus sequence is enriched in TDP-43 binding motifs, and the overexpression of wild-type TDP-43 diminishes the stability of Pol III transcripts and rescues IFN responses, whereas the RNA-binding mutant TDP-43 does not^44^. In addition, the RNase III Dicer can directly process *Alu* transcripts to downregulate their expression. Indeed, multiple studies revealed that Dicer depletion induces the accumulation of *Alu* RNAs, which promotes the development of geographic atrophy (GA), a form of age-related macular degeneration characterized by inflammation in the retinal pigment epithelium (RPE)^45,46^. Interestingly, upregulated *Alu* expression and the consequent cytotoxicity due to Dicer deficiency are mitigated by Pol III inhibition^45^. In addition, the authors found notable enrichment of *Alu* RNAs among J2-immunoprecipitated RNAs in GA patient samples that exhibited reduced Dicer levels, suggesting that Dicer might digest Pol III-transcribed IR*Alus*. In Dicer-deficient RPE cells, undegraded *Alu* RNAs activate the NLRP3 inflammasome and trigger TLR-independent MyD88 signaling, thereby promoting RPE cell death^46^. Kerur et al. further demonstrated that Dicer deficiency-induced inflammasome activation in RPE cells depends on cyclic GMP-AMP synthase (cGAS)-driven IFN signaling^47^. The authors suggested that *Alu* RNAs undergo reverse transcription to cDNA in the cytosol and that *Alu* cDNAs interact with cGAS, thereby facilitating the cytosolic release of mitochondrial DNA^48^. This event amplifies the activation of cGAS and induces IFNβ, consequently driving inflammasome activation and apoptosis. Overall, the immunogenic toxicity of the *Alu* element manifests in diverse manners, including *Alu* duplexes (mostly IR*Alus*) and *Alu* cDNAs (Fig. 1f).

### Gene regulation by IR*Alus*

In addition to their immunogenic roles, IR*Alus* play a pivotal role in gene regulation. *Alu* elements are ubiquitously dispersed within tens of thousands of gene bodies, giving rise to the broad transcription of embedded *Alu* and IR*Alus* as parts of Pol II-transcribed RNAs, including mRNAs^5,49^. Over the past decade, an increasing number of studies have reported the unique regulatory roles of embedded IR*Alus* in gene expression, ranging from circRNA biogenesis to mRNA nuclear retention. Below, we highlight the gene regulation by IR*Alus* through their structural characteristics and genomic locations.

#### Intronic IR*Alus*: alternative splicing and circular RNA biogenesis

One of the most well-understood mechanisms of *Alu*-mediated alternative splicing is *Alu* exonization. In particular, the minus strand of the *Alu* consensus sequence contains splice donor and acceptor sites, suggesting that the antisense *Alu* serves as a primary reservoir for generating alternative exons^49^. RNA folding of intronic *Alu* elements can also facilitate *Alu* exonization^50,51^. In this context, slow transcription can strengthen the intronic *Alu* RNA structure and cause nearby cryptic splice sites to resemble canonical splice sites, ultimately promoting *Alu* retention in mRNAs^50^. The incorporation of *Alu* exons in the coding region can introduce premature termination codons or trigger a frameshift in the encoded protein^52^.

**Fig. 2:**
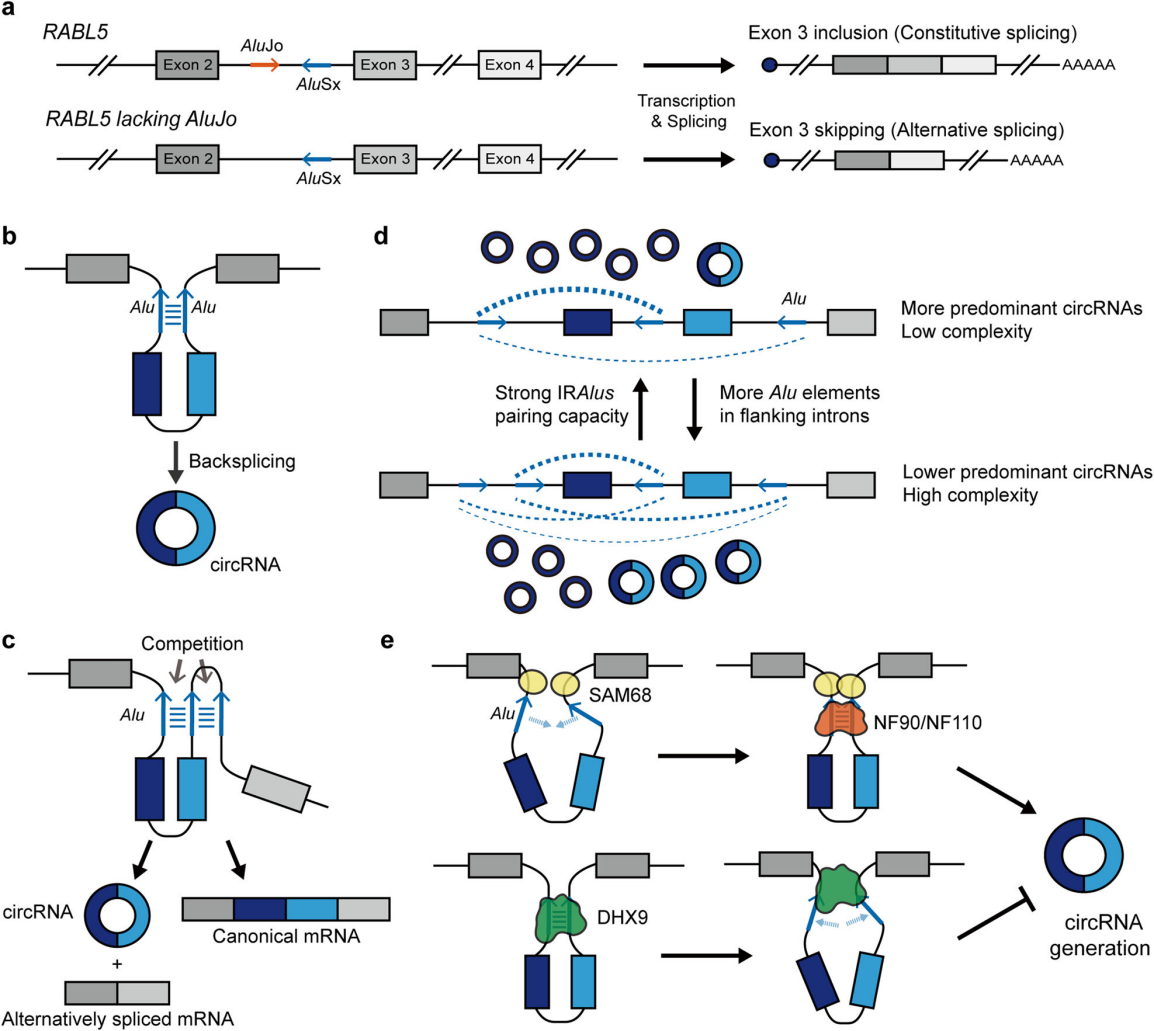
Intronic IR*Alus*-mediated alternative splicing and circRNA biogenesis. **a** An example of *Alu*-mediated alternative splicing in the *RABL5* gene. The antisense-oriented *Alu*Sx that is located just upstream of exon 3 suppresses exon 3 selection, leading to exon skipping. This negative impact mediated by *Alu*Sx is partly reversed by the sense-oriented *Alu*Jo situated in the same intron. **b** circRNA formation is facilitated by backsplicing, which is driven by RNA pairs formed by IR*Alus* across flanking introns. **c** RNA pairs formed by IR*Alu*s across neighboring introns compete with IR*Alu*s within the same intron, leading to competition between backsplicing for circRNAs and canonical splicing for linear RNAs. **d** Competition between IR*Alu*s across different pairs of flanking introns in the same gene locus promotes ABS, resulting in multiple circRNAs sharing the same backsplicing sites. **e** *Trans*-acting RBPs regulate circRNA biogenesis via direct interactions with intronic IR*Alus* flanking backspliced exons. SAM68 and NF90/NF110 positively regulate circRNA production by facilitating and stabilizing the formation of intronic IR*Alus*, while DHX9 represses circRNA production by destabilizing intronic IR*Alus*.

The splice sites within the antisense *Alu* can also regulate the alternative splicing of neighboring exons^53^. In the case of the *RABL5* gene, two *Alus* (*Alu*Jo in the sense orientation and *Alu*Sx in the antisense orientation) are inserted within the intron upstream of exon 3. Here, *Alu*Sx suppresses exon 3 selection, resulting in exon skipping^53^. Interestingly, this exon skipping event is partly counteracted by *Alu*Jo located in the same intron. The interaction between these two *Alus* forms a dsRNA structure, and the deletion of *Alu*Jo or increase in the distance between the two *Alus* enhances *Alu*Sx-mediated exon skipping, suggesting that intronic IR*Alus* formation mediates the delicate balance between canonical and alternative splicing of downstream exons (Fig. 2a).

One of the key splicing processes through which intronic IR*Alus* exert a significant impact is the generation of circRNAs via the promotion of backsplicing. Backsplicing ligates a downstream splice donor site with an upstream splice acceptor site, leading to the formation of covalently closed circular transcripts or circRNAs^54^. Typically, backsplicing competes with canonical splicing for the spliceosomal machinery but occurs less efficiently, resulting in fewer circRNAs than their linear counterparts^55^. Nonetheless, due to their circular structure, circRNAs are highly stable, and human cells express tens of thousands of circRNAs^56–58^. Numerous studies have demonstrated that RNA pairings formed by IR*Alus* across flanking introns bridge distal splice sites in close proximity to facilitate backsplicing^57–60^ (Fig. 2b). Considering that RNA pairs can also be formed by IR*Alus* within the same intron, which favors canonical splicing of linear RNAs, competition among multiple *Alu* elements for binding to each other can influence the relative efficiency of backsplicing (Fig. 2c).

Binding of different intronic *Alus* within a single gene can lead to the generation of multiple circRNAs that share the same backsplicing site through alternative backsplicing (ABS)^61^. Comprehensive transcriptome analysis across 90 human tissue samples revealed that ABS events are prevalent during circRNA biogenesis and account for approximately 84% of circRNAs^62^. Predominant circRNAs exhibit longer flanking introns and accommodate more *Alu* elements than other circRNAs in the same ABS event. In addition, the greater pairing capacity of IR*Alus* for flanking introns enables the corresponding circRNAs to outcompete other circRNAs, thereby establishing them as the predominant circRNAs (Fig. 2d). Human-specific *Alu* species exhibit a more robust pairing capacity than their mouse counterpart, B1 SINEs, resulting in a greater prevalence of circRNAs in humans than in mice^63^. For instance, survival motor neuron (*SMN*) genes exhibit significant enrichment of *Alu* elements, which occupy approximately 40% of the transcribed region of the *SMN*^64^. While the coding sequences of the *SMN* genes are highly conserved in mammals, these frequent *Alu* insertions result in a greater diversity of exon-containing circRNAs in humans^65^. Therefore, intronic *Alu* elements diversify human RNA landscapes through alternative splicing regulation.

In addition to *Alu-Alu* interactions, *trans*-acting RBPs are involved in the regulation of circRNA biogenesis by directly interacting with intronic IR*Alus* flanking backspliced exons. For example, nuclear factor 90 (NF90) and its 110 kDa isoform NF110 promote circRNA production by stabilizing the intronic IR*Alus* structure through direct binding to facilitate backsplicing^66^. The depletion of NF90 or NF110 results in widespread downregulation of nascent circRNA expression, which can be restored by reintroducing wild-type NF90 but not NF90 that lacks the dsRNA-binding motif. Furthermore, ATP-dependent DExH-Box Helicase 9 (DHX9) suppresses circRNA formation by binding and destabilizing intronic IR*Alus*^24^. Loss of DHX9 promotes the generation of a subset of circRNAs from *SMN* genes with a high density of *Alus*^65^. In contrast to that of DHX9, the Src-associated in mitosis 68 kDa (SAM68) protein can promote *SMN* pre-mRNA circularization by binding to flanking sites in *Alu*-rich regions of *SMN* introns^67^. The interaction between *Alu-*rich introns and SAM68 results in SAM68 dimerization, which may bring distantly located intronic IR*Alus* closer to promote *SMN* pre-mRNA circularization. Together, the coordinated interplay between *cis*-acting intronic IR*Alus* and multiple *trans*-acting RBPs regulates the biogenesis of circRNAs (Fig. 2e).

#### IR*Alus* in 3′ UTRs: Nuclear sequestration and mRNA decay

In addition to introns, 3′ UTRs contain a large number of IR*Alus* elements, which provide an additional layer of post-transcriptional gene regulation. The most well-known gene regulatory mechanism of interest associated with the 3′ UTR IR*Alus* is the nuclear sequestration of host mRNAs^13^. In the nucleus, inosine-containing RNAs are recognized by the paraspeckle protein non-POU domain-containing octamer binding (NONO)^68^. Considering that most A-to-I editing occurs on the IR*Alus* element, NONO recognizes IR*Alus* RNAs and can transport them to nuclear paraspeckles. Moreover, Chen et al. reported that hundreds of mRNAs containing IR*Alus* in the 3′ UTR undergo A-to-I editing events, and these IR*Alus*-containing mRNAs are sequestered in the nucleus in a NONO-dependent manner^13^.

**Fig. 3:**
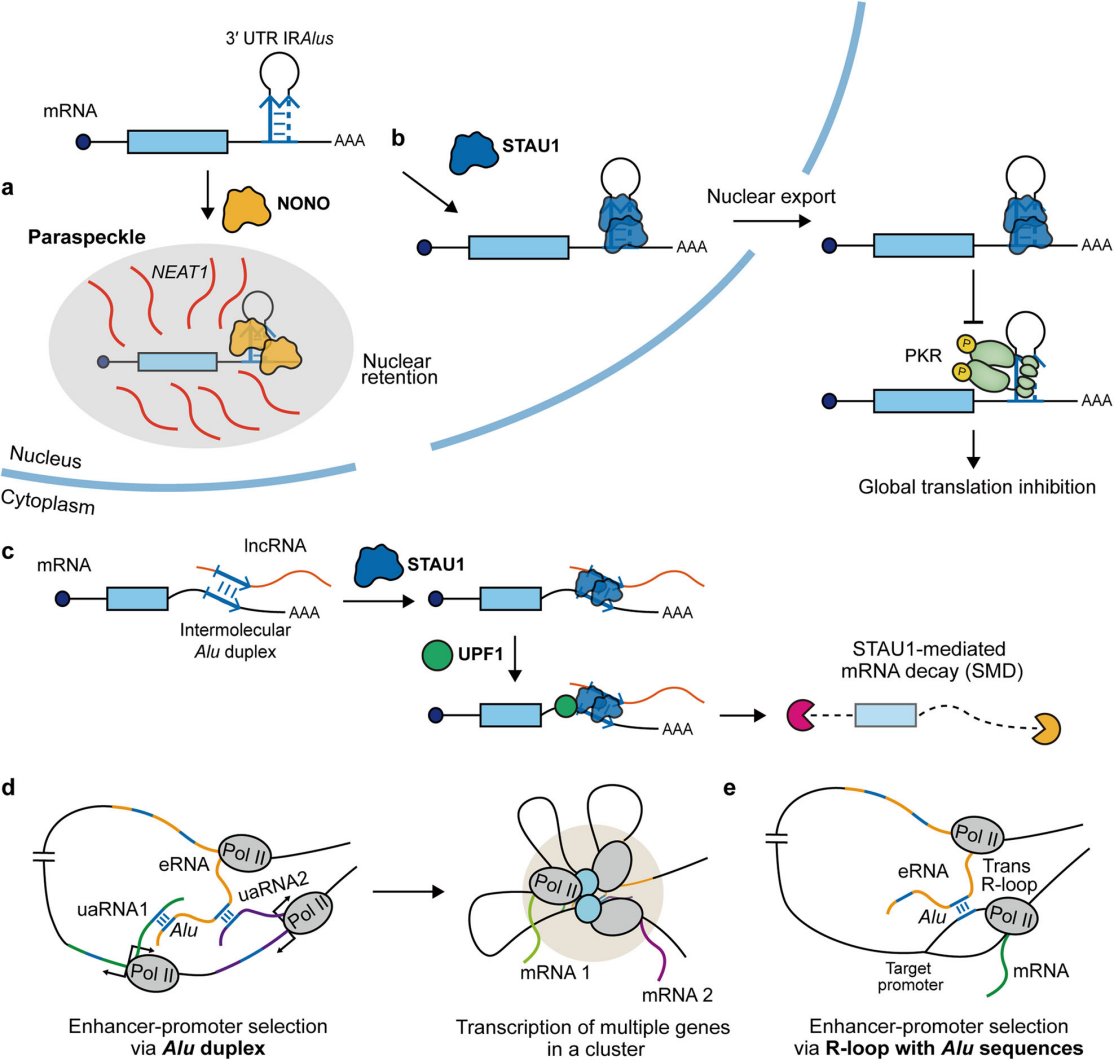
IR*Alus*-mediated gene regulation. **a** NONO recognizes 3′ UTR IR*Alus* in mRNAs and sequesters them in nuclear paraspeckles. **b** STAU1 facilitates the cytosolic export of IR*Alus*-containing mRNAs and enhances the translation of the corresponding mRNAs in the cytosol by preventing PKR binding. **c** STAU1 binds to the intermolecular *Alu* duplex and recruits the UPF1 RNA helicase to trigger SMD. **d** Long-distance enhancer-promoter interactions are facilitated by the formation of duplexes between the embedded *Alu* sequences in eRNAs and uaRNAs, which enables the transcription of multiple genes in the cluster. **e** R-loop formation through direct *Alu* RNA-DNA pairing facilitates the interaction between eRNAs and target promoters.

Paraspeckles are membrane-less nuclear organelles that primarily consist of two components: nuclear-enriched abundant transcript 1 (*NEAT1*), a long noncoding RNA (lncRNA), and several paraspeckle assembly proteins, including NONO^13,23^. The interaction between IR*Alus* and NONO results in the retention of mRNAs within the nucleus, subsequently leading to repression of protein translation in the cytosol (Fig. 3a). Hence, proper assembly of paraspeckles is essential for nuclear sequestration of mRNAs and subsequent gene silencing effects. A deficiency in the *NEAT1* lncRNA in embryonic stem cells results in the loss of paraspeckle assembly and the cytosolic release of IR*Alus*-containing mRNAs^23^. Additionally, the transcriptional suppression of the *NEAT1* lncRNA by coactivator-associated arginine methyltransferase 1 (CARM1) hinders the formation of paraspeckles and relieves IR*Alus*-mediated gene silencing^69^. CARM1 also methylates the coiled-coil domain of NONO to reduce its binding affinity to IR*Alus* to further free the 3′ UTR IR*Alus*-containing mRNAs from nuclear retention^69^. In pituitary cells, paraspeckle proteins and *NEAT1* expression follow a rhythmic circadian pattern, resulting in the circadian assembly of paraspeckles^70^. This circadian pattern leads to rhythmic nuclear retention of IR*Alus-*containing reporter mRNAs, indicating that nuclear retention and gene silencing by 3′ UTR IR*Alus* may play a role in regulating the circadian rhythm.

mRNAs with IR*Alus* in their 3′ UTR can escape nuclear retention by interacting with RBPs that compete with NONO for binding to IR*Alus*. The expression level of staufen1 (STAU1) is inversely correlated with NONO-IR*Alus* binding and is correlated with cytosolic export and translation of the corresponding mRNAs^25^. Notably, STAU1 also enhances the translation of IR*Alus*-containing mRNAs in the cytosol by preventing the binding of PKR^25^, which triggers global translational repression via eukaryotic translation initiation factor 2α (eIF2α) phosphorylation^71^ (Fig. 3b). Indeed, uncontrolled cytosolic release of IR*Alus*-containing mRNAs can lead to global translational suppression via PKR activation. While PKR and IR*Alus*-containing mRNAs are predominantly localized in the cytosol and nucleus, respectively, they can interact during mitosis when the nuclear envelope is disintegrated^18^. Subsequent PKR activation leads to global translational repression during mitosis. Similar nuclear sequestration of mRNAs was observed in the case of mouse *CTN-RNA*, which contains inverted repeats of B1 SINE elements with hyper A-to-I editing^72^. Interestingly, under stress, the inverted B1 repeats of *CTN-RNA* are removed from the 3′ UTR via alternative polyadenylation (APA), which leads to increased cytosolic export of RNA and increased translation of the encoded protein. This finding indicates that APA might be a key process that determines cell- or tissue-specific gene regulation by 3′ UTR IR*Alus* (see Future perspectives in the regulation and application of IR*Alus* for details)^13,72,73^.

Another important role of 3′ UTR IR*Alus* is the regulation of host mRNA stability. In a process known as STAU1-mediated decay (SMD), STAU1 binds to the 3′ UTR and recruits the up-frameshift suppressor 1 (UPF1) RNA helicase to trigger mRNA decay upon translation termination upstream of the STAU1-binding site (SBS)^74^. SMD regulates the expression of genes harboring SBSs during myogenesis, cutaneous wound healing, and adipogenesis^75–77^. In particular, SBSs may be formed via intermolecular base pairing between two RNA molecules containing complementary *Alu* elements (Fig. 3c). For example, the *Alu* element within cytosolic polyadenylated lncRNA, known as 1/2-SBS RNA, can form a dsRNA structure with another complementary element in the 3′ UTR of an SMD target mRNA^75^. In addition, the *Alu* element of the sprouty RTK signaling antagonist 4 intronic transcript 1 (*SPRY4-IT1*) lncRNA, which is associated with aggressive behavior and poor prognosis in human cancers, can bind to the 3′ UTR of the transcription elongation factor B subunit 1 (*TCEB1*) mRNA, which inhibits *TCEB1* expression to promote cell metastasis^78^. In summary, IR*Alus* in 3′ UTRs are key crosstalk hubs where several RBPs interact to post-transcriptionally regulate gene expression.

#### Transcriptional regulation by IR*Alus*

The gene regulatory function of IR*Alus* RNAs extends to transcription, where IR*Alus* act as a *trans-*acting factor. Traditionally, *Alu* DNA elements serve as enhancers that are regulated by H3K4me1 for tissue-specific regulation of gene expression^7^. A recent study revealed the novel role of *Alu* RNA duplexes in modulating long-range enhancer-promoter selectivity^79^. Enhancer-promoter interactions often occur over long distances. However, how enhancers find their cognate promoters has remained unclear. Interestingly, enhancers and promoters can be bidirectionally transcribed by Pol II, thereby generating enhancer RNAs (eRNAs) and promoter-antisense RNAs (uaRNAs), respectively^80^. Liang et al. employed RNA in situ confirmation sequencing, which maps RNA-RNA interactions, to explore enhancer-promoter connectivity using pairwise interactions between eRNAs and uaRNAs^79^. The authors constructed high-resolution enhancer-promoter RNA interaction maps in multiple cell lines and found significant enrichment of the *Alu* consensus sequence. Notably, enhancers containing *Alu* elements have a greater frequency of interaction with promoters than enhancers lacking *Alu* elements. The authors proposed a model in which long-distance enhancer-promoter looping is facilitated by the formation of duplexes between the embedded *Alu* sequences in eRNAs and uaRNAs. Ultimately, these *Alu* duplexes robustly direct an enhancer to its corresponding promoter, thereby establishing the specificity of enhancer-promoter interactions over extended genomic distances (Fig. 3d). Another study proposed a *trans*-acting R-loop model in which *Alu* elements potentially mediate the interaction between eRNAs and target promoters through direct RNA-DNA pairing^81^ (Fig. 3e). Collectively, these recent findings reveal an unprecedented role for *Alu* RNAs as regulators of enhancer-promoter selectivity.

### Regulation of IR*Alus* via RNA editing

RNA modifications are important regulatory mechanisms in RNA metabolism, including RNA stability, transport, splicing, and structure^82^. Among over 100 distinct types of RNA modifications, RNA editing stands out as it diversifies the cellular functionality of RNA molecules by altering their sequence^83^. The most common type of RNA editing is A-to-I editing, which is catalyzed by enzymes encoded by the adenosine deaminase acting on RNA (*ADAR*) gene family. Mammals express three ADAR family members (*ADAR1*, *ADAR2*, and *ADAR3*), with *ADAR1* accounting for the majority of editing events^84^. As discussed above, most A-to-I editing by ADAR1 occurs on IR*Alus* RNAs. Global analysis of ADAR1-RNA interactions in human cells using CLIP-seq and investigation of the ADAR1 editome revealed that ADAR1 primarily targets *Alu* RNAs^27,85^. In this section, we introduce the downstream effects of ADAR1-mediated A-to-I editing on the function of IR*Alus*.

#### Modulation of IR*Alus* immunogenicity by ADAR1

**Fig. 4:**
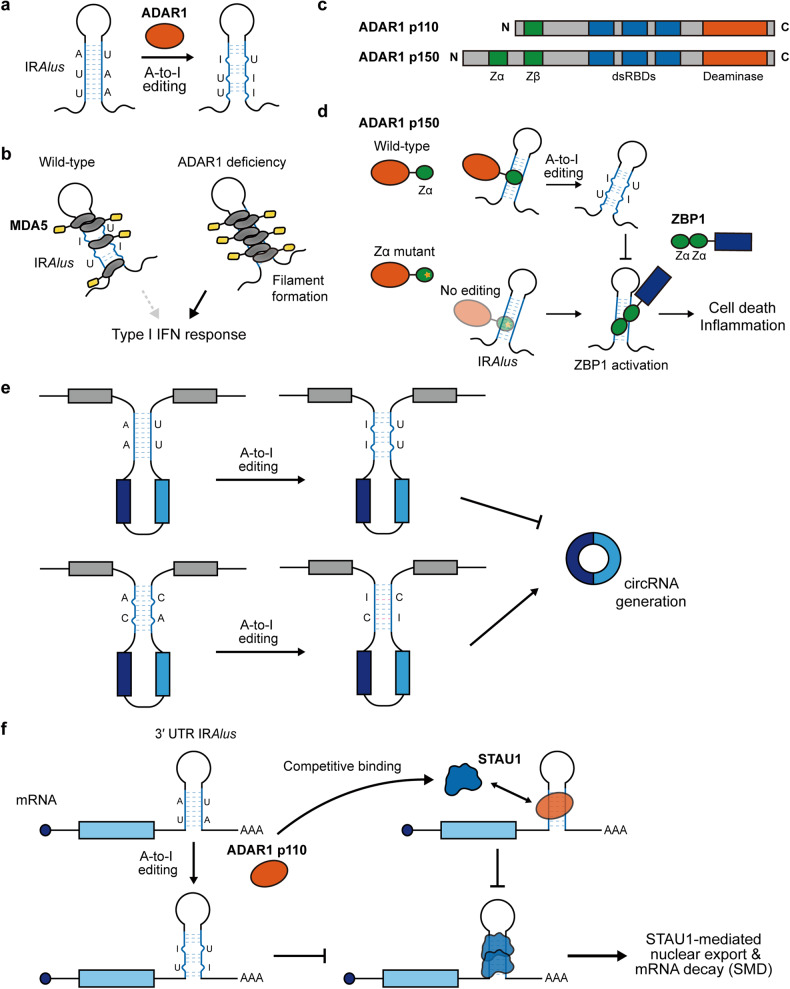
Modulation of IR*Alus* by ADAR1-mediated A-to-I editing. **a** A-to-I editing by ADAR1 reduces the structural integrity of dsRNAs by converting A-U pairs into the less stable I-U wobble. **b** Editing of IR*Alus* by ADAR1 diminishes the capacity of the RNA to activate MDA5 by reducing the efficiency of MDA5 filament assembly. Conversely, under ADAR1 deficiency, IR*Alus* become the primary ligands for MDA5, leading to MDA5 activation and the I-IFN response. **c** The two isoforms of ADAR1 include a constitutively expressed short nuclear isoform (p110) and a long IFN-inducible cytoplasmic isoform (p150). ADAR1 p150 contains the Zα domain in its extended N-terminus. **d** The interaction between the Zα domain-mediated ADAR1 with IR*Alus* in the Z-RNA conformation disrupts its secondary structure. In Zα mutant ADAR1 cells, unedited IR*Alus* are recognized by ZBP1 through its two Zα domains and trigger ZBP1-dependent cell death. **e** The dual roles of ADAR1 in modulating circRNA generation. A-to-I editing can destabilize or stabilize the dsRNA structure of IR*Alus* formed between flanking introns by creating I-U mismatches or correcting A-C mismatches to I-C pairs, respectively. **f** A-to-I editing affects the nuclear export of IR*Alus*-containing mRNAs by altering their binding to STAU1. In addition, ADAR1 prevents SMD of IR*Alus*-containing mRNAs through competitive binding with STAU1.

A key outcome of A-to-I editing is the alteration of dsRNA structural integrity by converting A-U pairs into less stable I-U wobble, resulting in decreased interactions with dsRNA sensors, such as MDA5 and PKR^86^ (Fig. 4a). Earlier studies revealed that editing of 3′ UTR IR*Alus* by ADAR1 resulted in reduced MDA5 activation by decreasing the efficiency of MDA5 filament assembly^19,87^. Indeed, under ADAR1 deficiency, 3′ UTR IR*Alus* become the primary ligands for MDA5 and can trigger MDA5 activation^19^ (Fig. 4b). Interestingly, one of the IFN-stimulated genes (ISGs), apolipoprotein L1 (*APOL1*), contains IR*Alus* in its 3′ UTR, which can activate MDA5 to upregulate its own expression through a positive feedback loop^88^. Moreover, MDA5 activation by unedited IR*Alus* can account for the frequent mutations in *ADAR1* in patients with autoinflammatory disorders, including AGS^89^. A recent exploration of the *cis-*RNAs quantitative trait locus revealed that common genetic variants linked to RNA editing levels are substantially enriched in genome-wide association study (GWAS) signals of common autoimmune and immune-related disorders^90^. Remarkably, diminished editing levels of IR*Alus* are apparent in GWAS-associated genetic variants; these findings underscore the fact that RNA editing is a key mechanism underlying the genetic risk for immune-related diseases.

ADAR1 is expressed as two isoforms, including a constitutively expressed short nuclear isoform (p110) and a long IFN-inducible cytoplasmic isoform (p150). While both ADAR1s can bind to canonical A-form dsRNA, the ADAR1 p150 isoform can bind to an unusual left-handed double helix called Z-RNA through the Zα domain in its extended N-terminus^91^ (Fig. 4c). Numerous studies have shown that mutations in the Zα domain of ADAR1 are commonly observed in patients with AGS, and the loss of the ADAR1/Z-RNA interaction triggers a spontaneous MDA5-dependent type I IFN response in both human and mouse cells^89,92–94^. Comprehensive analysis of A-to-I editing sites between wild-type and Zα mutant ADAR1 in human embryonic kidney 293 (HEK293) cells revealed that most of the sites less edited by the mutant were mapped to *Alu* elements^93^. Indeed, putative Z-RNA-forming sequences are present in *Alu* repeats, suggesting that the Zα domain of ADAR1 might be needed to efficiently edit *Alu* RNAs in the Z-RNA form^95^. Consistent with this thought, de Reuver et al. revealed that IR*Alus* are recognized by Z-DNA binding protein 1 (ZBP1) through its two Zα domains and stimulate ZBP1-dependent cell death in cells harboring the Zα mutant ADAR1^96^ (Fig. 4d). In summary, in addition to the recognition of dsRNA through its canonical dsRNA-binding domains, the Zα domain-mediated ADAR1 interaction with the IR*Alus* Z-RNA disrupts its secondary structure to maintain tolerance to endogenous dsRNAs.

A-to-I editing has received growing attention in cancer immunotherapies as increased expression of IR*Alus* by DNMTi treatment transforms the cells into the viral mimicry state. However, DNMTi treatment also promoted *ADAR1* transcription, which counteracted the increase in IR*Alus* expression via increased A-to-I editing. Considering this negative feedback by ADAR1, depletion of ADAR1 in combination with DNMTi treatment substantially limited the growth of colorectal cancer, whereas ADAR1 depletion alone had minimal effects^20^. Moreover, a similar strategy was employed for splicing dysregulation, where a combination of hnRNPC and ADAR1 deficiency synergistically triggered the induction of ISGs, underscoring the protective function of ADAR1 in mitigating IR*Alus*-mediated innate immune responses resulting from splicing dysregulation^26^. Since dysregulation of splicing occurs frequently in cancers^97^, such a strategy could be a promising approach to improve the efficacy of cancer immunotherapy.

#### Modulation of IR*Alus*-mediated gene regulation by ADAR1

A-to-I editing can also influence intronic IR*Alus*-mediated gene regulation. Studies have reported the association of ADAR1-mediated editing with circRNA formation through disruption of the dsRNA structure of intronic IR*Alus*^60,98^. Under ADAR1-deficient conditions, the enhanced stability of IR*Alus* pairing promotes backsplicing, resulting in increased circRNA expression^60^. In contrast, ADAR1 can promote circRNA generation in specific cases. A recent study reported that some A-to-I edits can stabilize dsRNA structures formed between flanking introns by correcting A-C mismatches to I-C pairs^98^. This is possible because ADAR catalyzes A-to-I editing on multiple adenosine residues in the vicinity rather than specifically on A-U paired adenosines. Overall, these findings highlight the importance of orchestrating the base pairing capacity of IR*Alus* through RNA editing, which introduces an additional layer of complexity to the circRNA transcriptome (Fig. 4e).

ADAR1 can also edit 3′ UTR IR*Alus* and affect the nuclear sequestration of host mRNAs. However, the effect of A-to-I editing during nuclear retention remains controversial. While a correlation between editing levels and nuclear retention levels exists^13^, it has been proposed that long dsRNAs stretch rather than inosine residues is needed^25^. For certain IR*Alus*-containing antiapoptotic genes, such as X-linked inhibitor of apoptosis protein (*XIAP*) and mouse double minute 2 (*MDM2*), ADAR1 influences the cytosolic export of mRNAs by altering the dsRNA structures and subsequently affecting the interaction with STAU1^99^ (Fig. 4f). Moreover, ADAR1 competes with STAU1 for binding to IR*Alus* and protects IR*Alus*-containing mRNAs from SBSs via SMD^100^ (Fig. 4f). Overall, the crosstalk between IR*Alus* and A-to-I editing by ADAR1 diversifies gene expression in cells.

### Future perspectives on the regulation and application of IR*Alus*

#### Biomolecular condensates

Recent studies on cellular dsRNAs have proposed potential regulatory mechanisms for IR*Alus*. Membrane-less biomolecular condensates, such as stress granules (SGs) and paraspeckles, are aggregates that are abundant in RNAs and RBPs^101^. Notably, SGs can prevent the excessive activation of dsRNA-induced innate immunity by both viral dsRNAs and increased self-dsRNAs in ADAR1 deficiency^102^. Although the specific identity of self-dsRNAs regulated by SGs has not yet been elucidated, we speculate that SGs modulate the immunogenicity of IR*Alus*. This hypothesis further calls for the investigation of the potential role of SGs in immune disorders associated with IR*Alus*. In addition, one recent study reported novel cytosolic condensates, referred to as dsRNA-induced foci (dRIFs), that are formed in response to increased levels of self- and non-self-dsRNAs^103^. These dRIFs contain dsRNAs and multiple RBPs, including PKR, ADAR1, STAU1, and DHX9. The authors proposed that dRIFs serve as sites of PKR activation by facilitating efficient PKR-dsRNA interactions. Notably, overexpression of an EGFP reporter mRNA with IR*Alus* in its 3′ UTR also induced the formation of foci that were colocalized with PKR, suggesting that the immunostimulatory activity of IR*Alus* might be modulated through dRIF formation. Deciphering the connection between biomolecular condensates and IR*Alus* will provide novel perspectives on the regulatory potential of IR*Alus*.

#### m^6^A modification

In addition to A-to-I editing, another prevalent RNA modification, N^6^-methyladenosine (m^6^A), can affect local dsRNA structures and the recognition of IR*Alus*. Strikingly, depletion of m^6^A triggers a detrimental immune response, resulting in hematopoietic failure and perinatal lethality during murine fetal development^104^. The responsible dsRNAs are predominantly derived from protein-coding regions and marked by high levels of m^6^A modification in their native states. Although determining the precise regulatory mechanism of m^6^A modification on dsRNA structures will require further investigation, m^6^A may function as a structural switch in these RNAs by disrupting the thermostability of base pairing^105^. Several studies have shown that m^6^A modification can indirectly affect IR*Alus* by modulating A-to-I editing, suggesting the potential for interplay between these two types of RNA modifications^106,107^. While the adenosines targeted by A-to-I editing or m^6^A modification are unlikely to overlap, ADAR1 exhibits an unfavorable association with m^6^A transcripts for further A-to-I editing, and suppression of m^6^A catalyzing enzymes results in a global increase in A-to-I editing^106^. This crosstalk could determine the editing status of 3′ UTR IR*Alus*, which might modulate IR*Alus* activity. Since other RNA modifications, such as m^1^A and m^5^C, have been shown to affect RNA secondary structures^82,108^, the interplay between different RNA modifications and the regulation of IR*Alus* needs to be explored.

#### Alternative polyadenylation

**Fig. 5:**
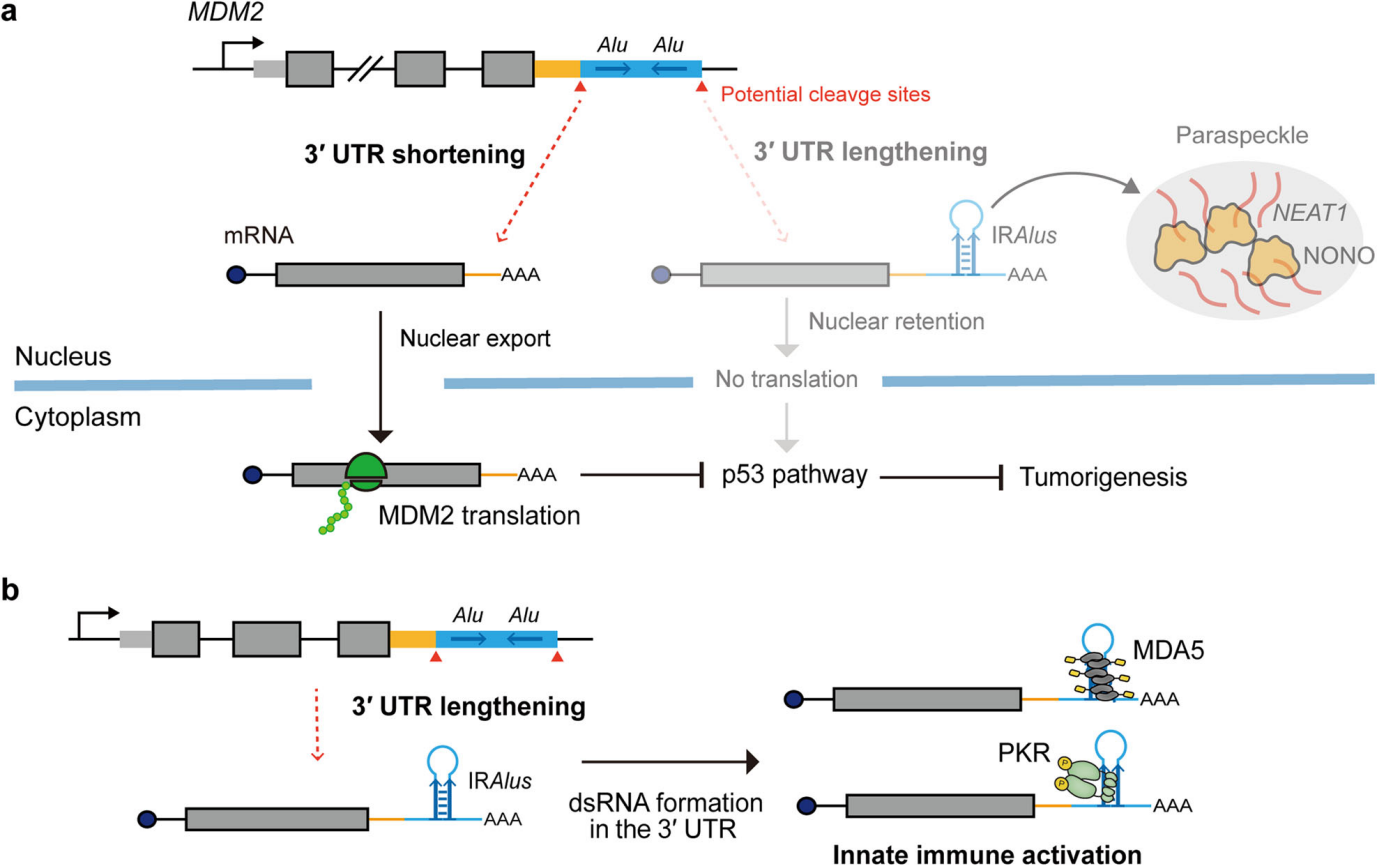
Regulation of IR*Alus* by APA. **a** IR*Alus* exclusion in *MDM2* through 3′ UTR shortening suppresses the p53 pathway. **b** 3′ UTR lengthening increases the overall expression of immunogenic self-dsRNAs via IR*Alus* incorporation and triggers innate immune activation.

Cell/tissue-specific alterations in 3′ UTR length present a novel perspective for regulating the functional landscape of IR*Alus*. A recent study by Ku et al. revealed that APA determines the inclusion or exclusion of IR*Alus* in 3′ UTR of hundreds of mRNAs, thereby globally affecting IR*Alus*-mediated gene regulation and essential cell signaling pathways^73^. For example, the authors revealed that global 3′ UTR shortening may contribute to tumorigenesis by excluding IR*Alus* in the 3′ UTR of *MDM2* mRNA, which leads to translational activation of *MDM2* and subsequent repression of the p53 signaling pathway (Fig. 5a). Conversely, they found that global 3′ UTR lengthening in neuronal progenitor cells facilitates 3′ UTR IR*Alus* inclusion, particularly in genes associated with the development of neurodegenerative diseases, including amyotrophic lateral sclerosis. Further investigations of cell/tissue-specific global APA changes and paraspeckle formation will advance our understanding of IR*Alus*-mediated gene regulation and its relevance to the pathogenesis of diverse human diseases.

In addition to IR*Alus*-mediated gene regulation, 3′ UTR length alteration may also modulate IR*Alus*-mediated innate immune activation. Recently, Dorrity et al. reported that extended 3′ UTRs in human neurons give rise to neuronal dsRNA structures, which results in high basal levels of self-dsRNAs and vulnerability to ADAR1 loss^109^ (Fig. 5b). The authors showed that the neuron-enriched genes *ELAVL2, ELAVL3, and ELAVL4* (*HuB*, *HuC*, and *HuD*) cooperatively increase the length of their 3′ UTR to trigger inflammation due to elevated dsRNA levels. Although the specific identity of immunogenic self-dsRNAs derived from extended 3′ UTRs in neurons has not been determined, the incorporation of IR*Alus* in longer 3′ UTRs might increase the burden of dsRNA. Similarly, in a study by Ku et al., 3′ UTR lengthening caused by the downregulation of cleavage stimulation factor subunit 2 (CSTF2) and its paralog CSTF2T expression, which are cleavage factors responsible for polyadenylation, promoted 3′ UTR IR*Alus* incorporation and subsequently resulted in inflammatory responses, including PKR activation^73^. Both studies proposed the regulation of 3′ UTR length as a novel strategy to modulate dsRNA-mediated immune responses.

#### Synthetic *Alu* RNA for cancer immunotherapy

A novel approach for cancer immunotherapy has emerged with the development of a synthetic RNA virus platform that consists of in vitro transcribed viral RNAs (vRNAs) encapsulated in lipid nanoparticles^110^. The intravenous administration of synthetic vRNAs to tumors facilitates virus replication and assembly, thereby stimulating immune cell infiltration and augmenting the activity of immune checkpoint inhibitors. Interestingly, a recent study demonstrated that nanoparticle delivery of synthetic *Alu* RNA also promotes antitumor immunity by activating RNA-sensing pathways^111^. The authors synthesized *Alu*Jb RNA (Left Arm), which can form intramolecular local dsRNA structures, and verified its immunostimulatory activity in vivo. Given that IR*Alus* exhibited enhanced immunostimulatory effects than did a single *Alu* due to their elongated dsRNA structures^18,19^, synthetic IR*Alus* RNAs may serve as more potent innate immune agonists. Current cancer immunotherapies leveraging the antitumor immunity of endogenous IR*Alus*, such as DNMTis and spliceosome inhibitors, are associated with a risk of unforeseen side effects owing to their global impact on the genome or transcriptome and variable responsiveness depending on the cell-specific degree of dsRNA induction^112,113^. Therefore, administering synthetic IR*Alus* (or *Alu)* RNAs can be an alternative strategy for precisely modulating dsRNA-mediated innate activation while minimizing potential side effects.

## Conclusion

High abundance and sequence similarity among *Alu* families allow multiple proximal *Alu* elements in an inverted orientation to form dsRNA structures called IR*Alus*. Numerous studies have attempted to unravel the physiological function of IR*Alus* and their roles in pathological conditions. These endeavors have significantly expanded our understanding of *Alu* elements. In this review, we highlight the multifaceted nature of IR*Alus*, with emphasis on their roles as self-immunogens and post-transcriptional gene regulators, and explain how IR*Alus* activity is regulated by RNA editing and interactions with RBPs. The recognition of IR*Alus* by dsRNA sensors and the downstream impacts are summarized in Table 1. In addition, the list of proteins modulating IR*Alus* activity and their corresponding functions are provided in Table 2.

**Table 1:** Summary of innate immune sensors for *Alu* elements and the downstream effects of their interaction.

*Alu* type	Innate immune sensors	Downstream impact and associated-diseases
IR*Alus* or *Alu* RNA duplex	MDA5	- Type I IFN response^19,92–94^ - Aicardi–Goutières syndrome (AGS)^19,30,92–94^
	PKR	- Global translation inhibition^18,27^ - Apoptotic cell death^27^
	RIG-I	- Type I IFN response^37,38,44^ - Inhibition of cell proliferation^37^ - Apoptotic cell death^38^
	ZBP1	- Inflammation^96^ - Necroptosis^96^
*Alu* cDNA	cGAS	- Type I IFN response and inflammasome formation^47,48^ - Geographic atrophy (GA)^47,48^

**Table 2:** Summary of the regulators of IR*Alus* and their functions.

*Alu* type	Regulators	Direct binding to *Alu* elements	Functions
All IR*Alus*	ADAR1	Yes	- Destabilizing base pairings of IR*Alus* via A-to-I editing^86^ - Preventing IR*Alus*-mediated innate immune responses^19,27,92–94,96^ - Modulating circRNA biogenesis^60,98^ - Modulating the localization and stability of IR*Alus*-containing mRNAs^99,100^
Pol III-transcribed *Alus*	TDP-43	Yes	- Regulating the expression, localization, and stability of Pol III-transcribed *Alus*^44^
	Dicer	Yes	- Preventing the accumulation of Pol III-transcribed *Alus*^45,46^
Intronic IR*Alus*	hnRNPC	Yes	- Preventing *Alu* exonization in pre-mRNAs^37^
	PRMT	No	- Preventing intronic IR*Alus* retention in mis-spliced mRNAs^38^
	DHX9	Yes	- Destabilizing intronic IR*Alus* and suppressing circRNA biogenesis^24^
	NF90/NF110	Yes	- Stabilizing intronic IR*Alus* and facilitating circRNA biogenesis^66^
	SAM68	No	- Binding to flanking sites to *Alu*-rich regions and facilitating circRNA biogenesis^67^
3′ UTR IR*Alus*	NONO	Yes	- Promoting nuclear sequestration of 3′ UTR IR*Alus-*containing mRNAs^13,23^
	STAU1	Yes	- Facilitating cytosolic export and translation of 3′ UTR IR*Alus-*containing mRNAs^25^ *-* Triggering SMD of 3′ UTR IR*Alus-*containing mRNAs^75^
	CARM1	No	- Reducing NONO binding affinity to 3′ UTR IR*Alus*^69^

By generating long dsRNAs, IR*Alus* are recognized as key endogenous activators of the innate immune response. Indeed, increased expression of IR*Alus* due to dysregulated epigenetics, as well as defects in RNA splicing and editing, is closely associated with the pathogenesis of immune-related disorders, including AGS^20,21,92–94^. Interestingly, recent studies have proposed the use of this immunogenicity of IR*Alus* in cancer therapy, in which small chemical inhibitors of DNA methylation or spliceosomes are used to transform the cancer cells into viral mimicry to induce apoptosis and sensitize cells to immunotherapy^20,21^. Inside the nucleus, IR*Alus* serve as a key post-transcriptional regulator of gene expression, depending on the genomic location. Intronic IR*Alus* contribute to the diversity of the human transcriptome by regulating circRNA biogenesis and alternative splicing^53,58,59^, while IR*Alus* in 3′ UTRs affect host gene expression by modulating the cellular localization and stability of mRNAs^13,75^. Recently, a study revealed the function of intermolecular IR*Alus* in transcription, where they mediate long-distance enhancer-promoter interactions^79^. Together, both intramolecular and intermolecular *Alu* duplexes exert their unique influence throughout the mRNA life cycle.

The activity of IR*Alus* is tightly modulated by ADAR1-mediated A-to-I editing, which melts base pairs and disrupts the secondary structure of IR*Alus*^19,27,87^. Emerging evidence suggests that IR*Alus* may exist in the Z-RNA conformation and require the Zα domain of ADAR1 p150 and ZBP1 to recognize and control these RNAs^92,93,96^. The involvement of the Z-RNA conformation in the interaction with specific RBPs and the potential cellular functions remain to be addressed. Additionally, IR*Alus* serve as a platform for the crosstalk of multiple types of RNA modifications. Moreover, recent studies have proposed that the functional landscape of IR*Alus* can be regulated by changes in 3′ UTR length through APA^73,109^. Given that RNA editing, modification, and APA exhibit cell line- and tissue-specific variability^114–117^, exploring tissue-specific IR*Alus* activity and their corresponding functions will augment our understanding and unravel the biological significance of highly abundant primate-specific *Alu* elements.
